# Recombination of the Phase-Variable *spnIII* Locus Is Independent of All Known Pneumococcal Site-Specific Recombinases

**DOI:** 10.1128/JB.00233-19

**Published:** 2019-07-10

**Authors:** M. De Ste Croix, K. Y. Chen, I. Vacca, A. S. Manso, C. Johnston, P. Polard, M. J. Kwun, S. D. Bentley, N. J. Croucher, C. D. Bayliss, R. D. Haigh, M. R. Oggioni

**Affiliations:** a Department of Genetics and Genome Biology, University of Leicester, Leicester, United Kingdom; b LMGM-CNRS UMR5100, CBI, Toulouse, France; c MRC Centre for Global Infectious Disease Analysis, Department of Infectious Disease Epidemiology, Imperial College London, London, United Kingdom; d Parasites and Microbes, Wellcome Sanger Institute, Hinxton, United Kingdom; Ohio State University

**Keywords:** DNA methylation, pneumococcus, phase variation, site-specific DNA inversion systems, site-specific recombination

## Abstract

Streptococcus pneumoniae is one of the world’s leading bacterial pathogens, causing pneumonia, septicemia, and meningitis. In recent years, it has been shown that genetic rearrangements in a type I restriction-modification system (SpnIII) can impact colony morphology and gene expression. By generating a large panel of mutant strains, we have confirmed a previously reported result that the CreX (also known as IvrR and PsrA) recombinase found within the locus is not essential for *hsdS* inversions. In addition, mutants of homologous recombination pathways also undergo *hsdS* inversions. In this work, we have shown that these genetic rearrangements, which result in different patterns of genome methylation, occur across a wide variety of serotypes and sequence types, including two strains (a 19F and a 6B strain) naturally lacking CreX. Our gene expression analysis, by transcriptome sequencing (RNAseq), confirms that the level of *creX* expression is impacted by these genomic rearrangements. In addition, we have shown that the frequency of *hsdS* recombination is temperature dependent. Most importantly, we have demonstrated that the other known pneumococcal site-specific recombinases XerD, XerS, and SPD_0921 are not involved in *spnIII* recombination, suggesting that a currently unknown mechanism is responsible for the recombination of these phase-variable type I systems.

**IMPORTANCE** Streptococcus pneumoniae is a leading cause of pneumonia, septicemia, and meningitis. The discovery that genetic rearrangements in a type I restriction-modification locus can impact gene regulation and colony morphology led to a new understanding of how this pathogen switches from harmless colonizer to invasive pathogen. These rearrangements, which alter the DNA specificity of the type I restriction-modification enzyme, occur across many different pneumococcal serotypes and sequence types and in the absence of all known pneumococcal site-specific recombinases. This finding suggests that this is a truly global mechanism of pneumococcal gene regulation and the need for further investigation of mechanisms of site-specific recombination.

## Abstract (precis)

Streptococcus pneumoniae is a leading cause of pneumonia, septicemia, and meningitis. The discovery that genetic rearrangements in a type I restriction-modification locus can impact gene regulation and colony morphology led to a new understanding of how this pathogen switches from harmless colonizer to invasive pathogen. These rearrangements, which alter the DNA specificity of the type I restriction-modification enzyme, occur across many different pneumococcal serotypes and sequence types and in the absence of all known pneumococcal site-specific recombinases. This finding suggests that this is a truly global mechanism of pneumococcal gene regulation and the need for further investigation of mechanisms of site-specific recombination.

## INTRODUCTION

Streptococcus pneumoniae is a major human pathogen responsible for pneumonia, septicemia, and meningitis, along with less severe infections such as otitis media and sinusitis. It is estimated that S. pneumoniae colonizes ∼50% of children under the age of 5 years in the United Kingdom (1). Although this colonization is largely asymptomatic, pneumococcal infections are the leading cause of lower respiratory infection morbidity and mortality globally (2). Phase variation (PV), the ability to reversibly change protein expression, is often controlled by switching genes on and off stochastically within bacterial populations. PV permits rapid adaptation to host environments and is therefore likely to contribute to both the asymptomatic spread of S. pneumoniae as well as the poorly understood switch between harmless colonizer and invasive pathogen.

In recent years, phase-variable (PV) type I restriction-modification (RM) systems, capable of changing HsdS protein expression through the movement of target recognition domains (TRDs), have been identified in many bacterial species (3). Rather than switching the expression of the type I RM system on and off, these PV changes, through DNA inversions, permit the expression of multiple *hsdS* genes in a bacterial population. Species with these systems include, but are not limited to, S. pneumoniae (4–8, 17), Listeria monocytogenes (9), Bacteroides fragilis (10), Enterococcus faecalis (11), Lactobacillus salivarius (12), and Streptococcus suis (13, 14). Our understanding of the biological relevance of these systems is limited despite being first identified in Mycoplasma pulmonis more than 20 years ago (15). Interestingly, nearly all of these PV type I RM systems contain an associated site-specific recombinase within their locus (3). This suggests that site-specific recombination may be a key mechanism facilitating the rearrangement of TRDs and, therefore, PV by methylation in a variety of bacterial pathogens.

SpnIII, also known as the *ivr* (16) or *cod* (6) locus, is a ubiquitous PV type I RM system of the pneumococcus (6, 7, 16, 17). While some strains with modified *spnIII* loci have been detected, no sequenced pneumococcal strain has been found without an *spnIII* locus. SpnIII is encoded by the genes SPD_0451 through SPD_0455 in D39 (accession number NC_008533.2) and the genes SP_0505 through SP_0510 in TIGR4 (accession number NC_003028.3). PV results in variation through six versions of SpnIII with differing S subunits and consequent recognition sequences and was observed to occur at rates of >1%. A previous analysis found differential gene expression patterns for each active *hsdS* gene orientation (17). Expression patterns were generated using non-phase-variable mutants where all but two of the TRDs had been removed. These mutants also exhibited differences in virulence in murine models of infection (17) and in opaque (OP)/transparent (TP) colony morphology, a phenotype previously associated with differences in the colonization and invasive potential of pneumococcal isolates (18). Multiple studies have linked specific *hsdS* expression states to a particular proportion of OP to TP colonies (6, 17, 19). Thus, SpnIII PV has a significant potential to impact the colonization or invasion phenotype during a clonal pneumococcal infection.

PV of SpnIII is driven by site-specific recombination between inverted repeat (IR) sequences. Site-specific recombination is mediated by enzymes that catalyze the cleavage and rejoining of DNA fragments independently of RecA and other homologous recombination machinery (20). Site-specific recombinases, the enzymes that facilitate these reactions, cut and religate at specific recognition sequences without ATP or the synthesis of any new DNA (21). These enzymes occur in a wide variety of bacteria and phages and enable the inversion and excision of DNA, depending on the presence of either inverted (inversion) or direct (excision) repeats (22, 23). Intragenomic recombination in bacteria can also be mediated by the universal recombinase RecA, and this process also has the potential to contribute to SpnIII PV. In the pneumococcus, RecA is required for homologous recombination enabling both the repair of DNA breaks and the integration of incoming DNA during natural transformation (24–26). In the pneumococcus, three different DNA loading complexes facilitate the loading of RecA onto DNA as part of three independent pathways of homologous recombination (27). First, the RecFOR complex loads RecA onto single-stranded DNA (ssDNA) gaps when replication forks stall (26, 28). Second, RexAB (a functional homologue of RecBCD) loads RecA onto regions with double-stranded DNA (dsDNA) breaks (29). Finally, the product of the transformation-dedicated recombinase loader gene *dprA* loads RecA onto incoming DNA during transformation, thereby facilitating incorporation into the chromosome when homologous regions of DNA are present (25, 27).

The mechanisms responsible for SpnIII PV have not been fully defined. Previous work in M. pulmonis demonstrated that a single site-specific recombinase was solely responsible for PV of two loci, including one type I RM system (30). Recently, Kwun et al. (31) reported that the direct-repeat-mediated shuffling of TRDs at the *tvr* locus of S. pneumoniae, encoding the type I RM system SpnIV, is enhanced but not exclusively controlled by the site-specific recombinase found within the locus. Similarly, variation in the SpnIII locus was found to occur in the absence of the site-specific recombinase (6, 32). Our aim was to determine whether site-specific, homologous recombination, or another process, drives the stochastic movement of TRDs between active and silent positions at the *spnIII* locus in S. pneumoniae D39. We show that TRD switching is independent of the universal recombinase RecA but is partially controlled by the site-specific recombinase CreX (also known as IvrR and PsrA), which is encoded within the *spnIII* locus.

(Parts of this work are included in the Ph.D. thesis of M. De Ste Croix, which can be accessed from the University of Leicester Research Archive.)

## RESULTS

**FIG 1:**
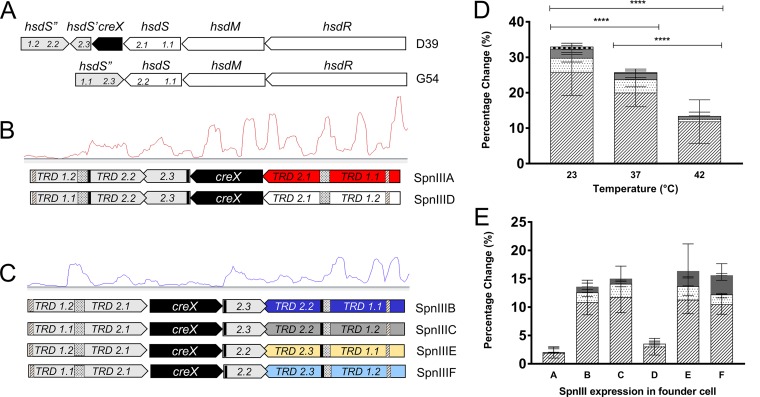
Active *hsdS* distribution in S. pneumoniae. (A) All sequenced pneumococcal isolates have an *spnIII* locus. However, in some strains, including G54 (GenBank accession number NC_011072), the locus appears to have undergone a deletion event; compared to D39, the G54 locus lacks the truncated *hsdS*′ gene and the *creX* recombinase and shows a duplication of target recognition domain (TRD) 1.1 with no TRD 1.2 (TRD numbering within the arrow of the *hsdS* genes). This results in G54 being limited to the active *hsdS* genes *hsdSB* and *hsdSE* only. (B and C) Due to recombination at the *spnIII* locus on the three inverted repeats, shown as hashed, dotted, and solid black boxes, the orientation of the *creX* recombinase can be aligned with *hsdR*, *hsdM*, and the active *hsdS* gene (B) or aligned with the unexpressed *hsdS*′ and *hsdS*″ genes (C). RNAseq data for an *hsdSA* (red)-expressing strain (B) and an *hsdSB* (blue)-expressing strain (C) were mapped in Artemis (45) using sorted BAM files. (D and E) Bars representing the mean percentages and SD of recombination within a minimum of 10 single colonies. Recombination events are expressed as a percentage of the total population by repeat. Recombination can occur on the 333-bp IR (dashed), the 85-bp IR (dotted), and the 15-bp IR (solid gray), and a small number are generated by >2 recombination events (checked). (D) S. pneumoniae D39 was grown at 23°C, 37°C, and 42°C to determine if temperature can impact the frequency of TRD shuffling. (E) Distribution of active *hsdS* genes in single colonies from each linage (lineages A to F). Statistical analysis was conducted using two-way analysis of variance (ANOVA). ****, *P* ≤ 0.0001.

The SpnIII restriction-modification system contains the classical type I RM system genes *hsdR* (restriction), *hsdM* (methylation), and *hsdS* (specificity), alongside two additional *hsdS* genes and a site-specific recombinase gene (*creX*) (Fig. 1A) (6, 17). Only one of the *hsdS* genes found within the locus is a complete gene and is transcribed (17); this gene is termed the active gene. The remaining two *hsdS* genes lack any transcriptional start sites and act as donors of target recognition domains (TRDs) (17). TRD shuffling is dependent on three IRs within the locus (Fig. 1B) and a on a core conserved target sequence of 10 bp (ATTATGGGAA) found within all IRs. We propose that these conserved 10 bp are essential for CreX-mediated recombination. Recombination frequencies were estimated for wild-type (WT) strain D39 and a wide range of other strains representing a range of different serotypes (see Fig. S1 in the supplemental material). Analysis of single colonies enables identification of the active gene present in the founder cell of the colony. As the growth of single colonies on an agar plate for 16 to 18 h is equal to approximately 20 to 22 generations, the percentage of cells that have undergone TRD shuffling on each of the three IRs can be determined as a proportion of the total population.

All mutant analysis was conducted with D39 and G54; however, to confirm that PV at the *spnIII* locus is widespread, 13 additional WT strains, representing 6 serotypes and 8 sequence types (STs), were analyzed (Fig. S1; Table S4). TRD shuffling was observed in all strains tested, including strains with an incomplete *spnIII* locus (G54 and BHN191). The rate of switching was variable between strains and differed greatly even between strains of the same ST (Fig. S1). The stock of D39 held within our laboratory collection predominantly expresses *hsdSE* (Fig. S1); therefore, all analysis in D39 has been restricted to colonies founded by *hsdSE* cells. The use of *hsdSE*-expressing cells allows inversions on all three repeats to be compared within a single colony. As the average number of switches within a single D39 *hsdSE* colony is dependent on the number of generations, colonies left on agar plates for increased periods of time will show higher levels of recombination. In a colony incubated for 16 h at 37°C, there are mean switching frequencies within the colony of 16.8% (standard deviation [SD], ±5.4%) on the 333-bp IR, 1.5% (SD, ±0.8%) (10-fold lower) on the 85-bp IR, and 4.1% (SD, ±1.9%) (3-fold lower) on the 15-bp IR.

To determine the impact of growth conditions on TRD shuffling, D39 was grown at 23°C, 37°C, and 42°C with 5% CO_2_ (Fig. 1D). TRD shuffling was significantly more frequent at 23°C than at 37°C (*P* < 0.0001) or 42°C (*P* < 0.0001). While growth at 42°C did not eliminate shuffling, inversions on the largest 333-bp repeat were reduced, as shown by the increase in the proportion of *hsdSA* in colonies founded by *hsdSE*-expressing cells from 11.8% (SD, ±6.2%) at 42°C to 19.9% (SD, ±3.9%) at 37°C and further still to 25.7% (SD, ±6.5%) at 23°C.

To investigate the impact on TRD shuffling of the site-specific recombinase CreX, also known as PsrA (32), transcriptome sequencing (RNAseq) analysis was performed on WT strains enriched for a single active *hsdS* gene. When in the same orientation as the *hsdR*, *hsdM*, and active *hsdS* genes, the CreX gene was expressed at a level 2.6-fold higher than when inverted and colinear with the silent *hsdS*′ and *hsdS*″ genes (Fig. 1B and C). Interestingly, the increased *creX* expression of *hsdSA* and *hsdSD* variants was associated with an 80% reduction in recombination at the 333-bp IR, 2.6% (SD, ±1.4%), compared to 11.6% (SD, ±2.3%) in the other four variants. Note that recombination at the short repeat is not quantified but is presumed to still be occurring in these variants (Fig. 1E). These results imply that CreX may block other forms of recombination at the locus.

**FIG 2:**
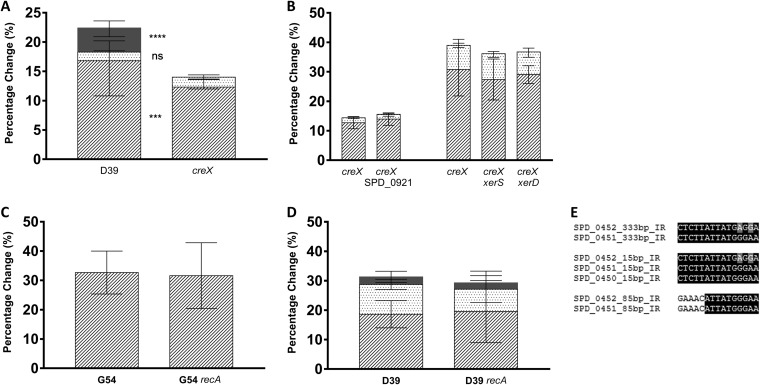
Site-specific and homologous recombination in SpnIII TRD switching. Bars represent the mean percentages and SD within a minimum of 10 *hsdSE* single colonies for each strain. Recombination can occur on the 333-bp IR (dashed), the 85-bp IR (dotted), and the 15-bp IR (solid gray), and a small number can be generated by >2 recombination events (checked). (A) The CreX recombinase is responsible for all switching on the smallest inverted repeat within the locus. In addition, there is significantly less recombination at the 333-bp IR in this strain (*P* = 0.0007); the 85-bp IR is unaffected by the loss of CreX. (B) Double-knockout mutants lacking *creX* and each of the other known, functional site-specific recombinases within the D39 genome (33) were tested for their impact on TRD shuffling. No significant differences were found between the parent *creX* mutant and the double-knockout mutants. (C and D) The involvement of homologous recombination to facilitate TRD switching was tested in two different genetic backgrounds: D39 (C) and G54 (D). The active gene distribution was not found to be significantly different between the WT and *recA* mutants. G54 has an alternative *spnIII* locus lacking TRDs 1.2, 2.1, and 2.1 and the *creX* recombinase gene. The absence of the *creX* gene in G54 shows that the absence of RecA does not lead to a significant difference in either a *creX* WT or a *creX* mutant background. Statistical analysis was conducted using two-way ANOVA. ***, *P* ≤ 0.001; ****, *P* ≤ 0.0001; ns, not significant. (E) The conserved 10-bp consensus in the three inverted repeats within the *spnIII* locus of the site-specific CreX recombinase.

To examine the specific role of CreX, we generated a frameshift mutant that truncated the CreX protein at amino acid 6 without altering the size of the gene. TRD shuffling in single colonies of the *creX* mutant (MRO633) were compared to that of single colonies of the WT D39 strain. In the absence of the CreX recombinase, shuffling between the smallest 15-bp IR was no longer observed (i.e., no *hsdSB* variants were generated in *hsdSE*-founded cells) (Fig. 2A). This is in accordance with the hypothesis that site-specific recombination at the conserved 10-bp sequence present in all three IRs (ATTATGGGAA) is strictly CreX dependent. Shuffling to the *hsdSA* variant was also reduced from 15.8% (SD, ±5.4%) to 12.7% (SD, ±2.0%) (*P* = 0.0007), showing that CreX may be partially but not fully responsible for these events. Shuffling on the 85-bp IR (*hsdSE* to *hsdSD*) was unaffected (*P* = 0.9). The lack of an impact on the 85-bp IR may be explained by the difference in the 5 bp immediately upstream of the conserved 10-bp sequence (Fig. 2E); in the 333-bp and 15-bp IRs, the sequence appears to be conserved as CTCTT, but this is not the case for the 85-bp IR (GAAAC). The observation that CreX is not the sole mechanism of control for TRD shuffling is supported by evidence from the analysis of strains G54 (Fig. 1A) and BHN191 (Fig. S1), both of which lack the CreX recombinase and at least one TRD. Both G54 and BHN191 display frequencies of recombination between the TRDs present that are similar to those observed on the 333-bp IR of D39 (Fig. S1). It is unlikely that these strains undergo recombination at the 15-bp IR in the absence of the CreX recombinase.

Double-knockout mutants of each of the other known, functional site-specific recombinases within the D39 genome (33) were tested to determine whether multiple site-specific recombinases controlled TRD shuffling at the *spnIII* locus. All double knockouts contain a *creX* frameshift along with the knockout of one other site-specific recombinase. Analysis of single colonies from double-knockout strains showed that site-specific recombinases SPD_0921, XerS, and XerD had no impact on *spnIII* TRD shuffling (Fig. 2B), ruling out the possibility of a cooperative interaction of these recombinases with CreX in a dual-site-specific recombinase mechanism such as that observed in the Hin locus of *Salmonella* (23). In addition to SPD_0921, XerS, and XerD, there is a truncated recombinase (SPD_1013) that is expected to be nonfunctional; a mutant for this gene has not been tested. The D39 genome was searched for homology to known serine and tyrosine recombinase genes, and no additional genes were found. Single knockouts of these site-specific recombinases were also analyzed and shown to have no significant impact on TRD shuffling (Table S5). Due to the impact of environmental conditions such as incubation time and temperature, each experimental analysis includes a paired WT or parental strain grown under the same conditions. As a result of these environmental variations, multiple strains can be found repeated in Table 2 with differing results. Differences in recombination are determined only between strains incubated at the same time under the same conditions.

In addition to testing site-specific recombination, we generated mutants in 10 recombination genes encompassing a variety of homologous recombination pathways (Table 1). No alterations in the rates of recombination were detected for any of the IRs by any of these mutations (Table 2), demonstrating that both RecA- and RecFOR-mediated recombination are not involved in *spnIII* PV. To further investigate the role of RecA, a *recA* mutant was constructed in strain G54 which has an incomplete *spnIII* locus lacking TRDs 1.2 and 2.1 (with a duplicated TRD 1.1) and the CreX recombinase (Fig. 1A). In this strain, recombination at the *spnIII* locus occurs in a CreX-independent manner such that it is capable of producing only the active genes *hsdSB* and *hsdSE* as a result of recombination at one or both of the 85-bp and 333-bp repeats. Thus, this strain provides a useful model for determining the level of non-site-specific recombination. As in D39, no significant differences were observed in the frequencies of recombination for the G54 background between active *hsdS* genes in *recA* mutants and the parental strain (Table 2).

**TABLE 1:** Mutant strains used in this study

Strain	Mutant genotype	Gene(s)	Reference
MRO633	D39 *creX*	SPD_0452	This work
MRO639	D39 *creX* SPD_0921::*aad9*	SPD_0452, SPD_0921	This work
MRO652	D39 *creX xerD*::*aad9*	SPD_0452, SPD_1657	This work
MRO660	D39 *recU*::*aad9*	SPD_0337	This work
MRO661	D39 *spoJ*::*aad9*	SPD_2069	This work
MRO669^*a*^	D39 *recA*::*cat*	SPD_1739	35
MRO688	D39 *uvrA*::*aphIII*	SPD_0176	This work
MRO796^*a*^	G54 *recA*::*cat*	SPG_1849	35
MRO797	D39 *creX xerS*::*aad9*	SPD_0452, SPD_1023	This work
MRO810^*a*^	D39 *creX recA*::*cat*	SPD_0452, SPD_1739	35
MRO811^*a*^	D39 *ssbB*::*aad9*	SPD_1711	46
MRO812^*a*^	D39 *recN*::*aad9*	SPD_1062	27
MRO813^*a*^	D39 *recF*::*ermB*	SPD_2054	26
MRO814^*a*^	D39 *recR*::*aphIII*	SPD_1485	26
MRO815^*a*^	D39 *hexA*::*ermB*	SPD_1903	47
MRO816^*a*^	D39 *recG*::*cat*	SPD_1507	48

a The strain was generated by transformation of genomic DNA from the listed reference strain.

**TABLE 2:** Mean percentages of recombination by repeat in mutant and wild-type strains^*a*^

Strain	333-bp repeat		85-bp repeat		15-bp repeat	
	Mean % recombination ± SD	*P* value	Mean % recombination ± SD	*P* value	Mean % recombination ± SD	*P* value
D39	15.8 ± 5.4		1.2 ± 0.8		4.1 ± 1.9	
D39 *creX*	12.7 ± 2.0	0.0007	1.7 ± 0.6	NS	0.0 ± 0.0	<0.0001

D39 *creX*	12.3 ± 2.0		1.7 ± 0.6		0.0 ± 0.0	
D39 *creX* SPD_0921::*aad9*	13.2 ± 2.1	NS	1.6 ± 0.6	NS	0.0 ± 0.0	NS

D39 *creX*	30.8 ± 8.9		8.7 ± 1.2		0.0 ± 0.0	
D39 *creX xerS*::*aad9*	27.4 ± 7.0	NS	8.0 ± 2.7	NS	0.0 ± 0.0	NS
D39 *creX xerD*::*aad9*	29.1 ± 3.0	NS	7.6 ± 1.2	NS	0.0 ± 0.0	NS

D39	10.8 ± 4.4		1.5 ± 0.6		3.1 ± 2.7	
D39 *ssbB*::*aad9*	9.8 ± 2.7	NS	1.6 ± 0.8	NS	2.6 ± 0.4	NS
D39 *recN*::*aad9*	10.9 ± 2.1	NS	1.5 ± 0.7	NS	2.2 ± 1.1	NS
D39 *recF*::*ermB*	10.4 ± 2.0	NS	1.8 ± 0.5	NS	3.7 ± 5.2	NS
D39 *recR*::*aphIII*	8.8 ± 6.3	NS	1.3 ± 0.6	NS	3.3 ± 0.7	NS
D39 *recG*::*cat*	8.0 ± 1.6	NS	1.4 ± 0.4	NS	2.7 ± 0.7	NS
D39 *hexA*::*ermB*	11.3 ± 1.8	NS	1.2 ± 0.7	NS	2.4 ± 0.8	NS

D39	19.7 ± 5.2		2.2 ± 1.4		2.4 ± 1.3	
D39 *spoJ*::*aad9*	22.6 ± 4.3	NS	4.4 ± 1.7	NS	2.9 ± 1.1	NS

D39	16 ± 8.1		1.4 ± 1.5		4.4 ± 0.7	
D39 *recU*::*aad9*	15.4 ± 2.9	NS	3.3 ± 1.3	NS	4.6 ± 2.9	NS

D39	25.1 ± 7.7		8.9 ± 2.2		2.8 ± 0.9	
D39 *uvrA*::*aphIII*	30.9 ± 7.3	NS	6.8 ± 0.6	NS	1.7 ± 0.9	NS

D39	18.6 ± 4.6		10.1 ± 1.7		2.6 ± 1.9	
D39 *recA*::*cat*	19.5 ± 10.5	NS	7.6 ± 4.6	NS	2.2 ± 4.0	NS

G54^*b*^	36.6 ± 1.9					
G54^*b*^ *recA*::*cat*	37.4 ± 2.4	NS				

a NS, not significant.

b This strain lacks TRDs 1.2 and 2.1.

**FIG 3:**
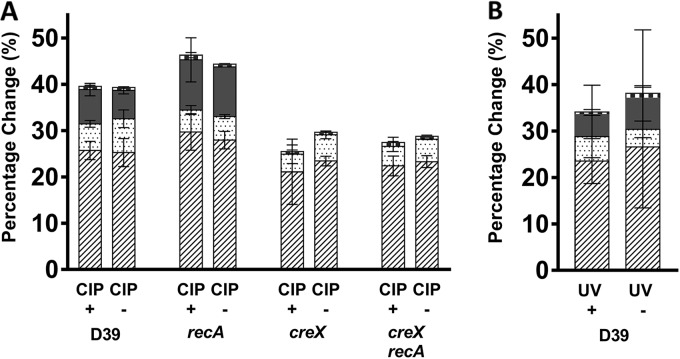
Impact of DNA damage on *spnIII* TRD switching. Bars represent the mean percentages and SD within a minimum of 10 *hsdSE* single colonies for each strain. Recombination can occur on the 333-bp IR (dashed), the 85-bp IR (dotted), and the 15-bp IR (solid gray), and a small number can be generated by >2 recombination events (checked). (A) D39, D39 *creX*, D39 *recA*, and D39 *creX recA* were exposed to a sub-MIC dose of ciprofloxacin, and after 16 to 18 h of growth, colonies were analyzed for recombination events. (B) D39 was exposed to a sublethal dose of UV, and recombination events in 20 single colonies were compared to those of an unexposed control. Significance was tested using two-way ANOVA.

To further test whether RecA or other DNA repair pathways are involved in *spnIII* TRD shuffling, we tested strains under conditions known to induce DNA damage (Fig. 3). The D39 WT, *creX* frameshift mutant, *recA* mutant, and *creX recA* double mutant strains were grown with and without a subinhibitory concentration (1/2 the MIC value) of ciprofloxacin (CIP) (Fig. 3A). Strains were grown overnight for 16 h (18 to 20 generations); *recA* mutants were incubated for 24 h (16 to 18 generations) due to their low growth rate. For colonies grown with and without CIP, no significant differences were observed in the percentages of active *hsdS* genes in any strain, suggesting that CIP has no impact on TRD shuffling at the *spnIII* locus. Additionally, we tested the impact of UV irradiation on *spnIII* recombination (Fig. 3B). An exponentially growing culture was split, with one half receiving a sublethal UV dose before plating. Approximately 90% of exposed cells were killed following UV exposure (data not shown), but surviving cells showed no significant variation in TRD shuffling compared to unexposed cells. Both the ciprofloxacin and UV exposure observations confirm that SpnIII PV involves RecA-independent recombination.

To directly identify proteins with a role in *spnIII* TRD shuffling, we generated a biotin-labeled synthetic 1.2-kb double-stranded DNA fragment containing one 333-bp repeat and two 15-bp repeats in an inverted orientation. This fragment was incubated with pneumococcal whole-cell lysates from an exponential-phase culture stimulated with competence-stimulating peptide 1 (CSP1), followed by purification of DNA-bound proteins using streptavidin-coated beads. Bound proteins were analyzed by mass spectrometry. Most of the DNA binding proteins were encoded by essential genes (Table S6), except for the gene for the DNA repair protein UvrA. Based on these data, we generated a *uvrA* mutant, but again, inactivation of this protein had no significant impact on *spnIII* TRD shuffling (Table 2).

## DISCUSSION

Our findings indicate that SpnIII TRD shuffling is partially but not wholly controlled by the site-specific recombinase CreX (Fig. 2); however, we have demonstrated that the process is independent of other site-specific recombinases in the pneumococcal genome (33). Interestingly, the frequency of inversions is linked to growth temperature, in line with previous suggestions that PV of the SpnIII locus may offer an advantage in adaptation to different environments (6, 7, 16, 17). The presence of multi-*hsdS* systems in other pathogenic bacteria (9–14) may also prove to be a temperature-regulated method of environmental adaptation.

As the CreX enzyme is encoded within the majority of S. pneumoniae *spnIII* loci and is highly conserved between strains, our findings strongly suggest that it is a major regulator of SpnIII PV across the pneumococcus genus. Our initial expectation, that all TRD shuffling was controlled by CreX, is in line with previous observations in M. pulmonis (30) and B. fragilis (10). However, our finding that this is not the case aligns with recently reported data on the ability of these systems to recombine independently of their locus-encoded site-specific recombinase (6, 31, 32). Our analysis of strains (see Fig. S1 in the supplemental material) naturally lacking the CreX recombinase (G54 and BHN191) confirms that a locus-encoded site-specific recombinase is not essential to the process.

Surprisingly, the highest levels of CreX expression (Fig. 1) correspond with the lowest frequency of TRD shuffling on the longer repeats (i.e., in colonies founded by cells with active *hsdSA* and *hsdSD* genes), suggesting that it may not simply be the quantity of the CreX recombinase found within the cell that facilitates ongoing DNA inversions. One potential explanation for this decrease is that the CreX recombinase may form complexes with the conserved 10-bp target sequence within the repeats, thereby reducing or preventing the binding of other proteins that may play a part in TRD shuffling on the 85- and 333-bp IRs. The abolition of recombination at the smallest 15-bp IR indicates that the core target site for the CreX recombinase is the conserved core 10-bp sequence found in all three IRs (Fig. 2E); however, the 5 bp immediately upstream also appear to play a role, facilitating recombination at the 333-bp but not the 85-bp IR. This is supported by the work of Li et al. (32), who demonstrated that there was either a complete loss or a significant impairment of 15-bp-mediated inversions when single nucleotides of the 15-bp sequence were modified. This target size is similar to those of other site-specific recombinases such as Cre and the *loxP* site, both of which contain a core 13-bp sequence (34).

In the absence of CreX, shuffling of the 15-bp inverted repeats was abrogated but still occurred on the longer repeats (Fig. 2A). This complexity suggested the possibility of a dual-site-specific recombinase mechanism such as the one seen in the *Salmonella* flagellar locus (23). At this locus, the majority of DNA inversions are carried out by the Hin recombinase. A much lower frequency of inversions occurs in the absence of Hin and is mediated by the Fin recombinase, which is located elsewhere in the genome (23). Using recombination at the Hin locus as a model, we hypothesized that *hsdS* inversions may be mediated by a second site-specific recombinase within the pneumococcal genome. All functional site-specific recombinases were deleted from the genome as single mutants, as well double-knockout mutants, where all strains lacked the CreX recombinase to test for redundancy. Our analysis showed that no other site-specific recombinases in the D39 genome (33) have any impact on TRD shuffling at either the short or long repeats of the *spnIII* locus (Table 2; Table S5).

After ruling out the CreX recombinase as the sole mechanism of control of *hsdS* inversions, we explored the possibility that the cell’s homologous recombination machinery was partially responsible for TRD shuffling. There are several different homologous recombination pathways in the pneumococcus, including RecFOR and the genetic transformation pathway (26, 29, 35, 36). The universal recombinase RecA is crucial for both of these pathways; therefore, we explored whether strains lacking RecA were still capable of TRD shuffling. If RecA is required for DNA inversions to occur at the *spnIII* locus, a *recA* mutant would show a reduced level of *hsdS* inversions. We found that this was not the case, even when DNA damage was induced to stimulate repair pathways. It is known that ciprofloxacin-induced recombination is RecA dependent (37). The observation that the D39 WT and the D39 *recA* mutant strains show no differences when grown with and without a subinhibitory concentration of ciprofloxacin therefore confirms our hypothesis that *hsdS* inversions are RecA independent. In addition to testing RecA-mediated pathways, a *hexA* mutant confirmed that the mismatch repair pathway is also not involved.

RecA-independent recombination at large direct repeats has been shown to occur at low but detectable levels in Escherichia coli when sufficient sequence homology is present (38). The frequency of RecA-independent recombination can be significantly increased at shorter regions of homology (80 and 200 bp) when single-stranded exonucleases are no longer present (38). This provides evidence that under the correct conditions, high levels of RecA-independent recombination are not only achievable but a regular occurrence (38) and are likely controlled by the physical proximity of DNA sequences (40). The mechanism behind RecA-independent recombination remains unknown, even in E. coli; however, it has been proposed that DNA replication forks are required (38). The E. coli model showed high levels of RecA-independent recombination at both 80 and 200 bp and could therefore provide a suitable explanation for how TRD shuffling is controlled at the *spnIII* locus independently of site-specific recombination.

Recent work by Amarh et al. (41) has shown for E. coli that the endonuclease SbcCD is capable of DNA cleavage at specific sites to repair double-stranded breaks between sister chromosomes during DNA replication. SbcCD efficiently cuts just one of the sister chromosomes, initiating the repair of the double-stranded break using the intact copy. The subsequent repair process requires the RecABCD complex (41) and hence cannot fully explain the RecA-independent recombination observed at the *spnIII* locus in S. pneumoniae. Nevertheless, there are homologues of these genes in S. pneumoniae, and so this mechanism offers an attractive avenue for future work with the possibility that exonucleases could play a role in the ongoing DNA breakage and repair after inversion.

A key outcome of the complex nature of the control of TRD shuffling at the *spnIII* locus is that switching between each variant type occurs at different rates and could exhibit differential regulation by DNA replication or nonreplicative enzymes. The lack of recombinase cooperation in the ongoing site-specific recombination of *hsdS* genes suggests that there are proteins in the pneumococcal genome that are capable of generating DNA inversions that have yet to be identified and categorized. The temperature-dependent phenotype that we have observed is likely to play a role in better understanding and working with these multi-*hsdS* systems in the future and has the potential to explain the adaptability of some bacterial pathogens to a wide range of environmental temperatures. It is clear that there is a multifaceted control mechanism behind *hsdS* inversions in the SpnIII system. This may have phenotypic consequences, in particular where alternative SpnIII methylation patterns in a bacterial population impact adaptability and survival in challenging host environments.

## MATERIALS AND METHODS

### Growth and storage of bacterial strains.

S. pneumoniae strains were grown in liquid cultures in either BBL Trypticase soy broth (TSB) (Becton, Dickinson, USA) or brain heart infusion (BHI) broth (Oxoid, UK). For growth on solid medium, BBL Trypticase soy agar (TSA) or BHI agar (BHIA) supplemented with 3% (vol/vol) defibrinated horse blood (Thermo Scientific, UK) was used. Plates were incubated at 37°C with 5% CO_2_ for 16 to 18 h unless otherwise stated. Where appropriate, medium was supplemented with the following antibiotics at the indicated concentrations: ciprofloxacin at 0.25 μg/ml (0.016 μg/ml for *recA* mutant strains), kanamycin at 500 μg/ml, spectinomycin at 200 μg/ml, chloramphenicol at 10 μg/ml, streptomycin at 500 μg/ml, and erythromycin at 10 μg/ml.

### Transformation of S. pneumoniae.

BHI broth was inoculated with a 1:100 dilution of a frozen culture and then incubated at 37°C with 5% CO_2_ until an optical density at 590 nm (OD_590_) of 0.03 to 0.05 was reached. Cells were then diluted 1:10 into BHI broth supplemented with 0.1% (vol/vol) 1 M CaCl_2_, 0.2% (vol/vol) glucose, and 0.4% (vol/vol) bovine serum albumin (BSA) (Sigma, UK), known as BHI-CTM, and incubated at 37°C for 60 min. Two hundred microliters of cells was incubated with a minimum of 50 ng of DNA and 0.625 ng/μl of the competence-stimulating peptide CSP1 (Inbios, Italy). Cells were incubated for 45 min before plating with appropriate antibiotics onto BHI agar containing 3% (vol/vol) horse blood. Plates were incubated overnight at 37°C with 5% CO_2_. All mutants were confirmed by PCR and Sanger sequencing.

### Generation of mutants.

All mutant strains were created by the transformation of PCR-generated fragments or extracted genomic DNA (where strains were supplied by the laboratory of Patrice Polard) (Table 1). For PCR-generated fragments, approximately 500 bp up- and downstream of the gene to be deleted were amplified using primers (see Table S1 in the supplemental material) with 20-bp tails complementary to either an *aad9* (aminoglycoside *O*-nucleotidyltransferase) spectinomycin cassette, a Janus kanamycin cassette (*rpsL* [ribosomal protein S12] and *aphIII* [aminoglycoside *O*-phosphotransferase]) (42), or a chloramphenicol acetyltransferase cassette (*cat*) (39). Reamplification of the flanking regions with the appropriate cassette produced constructs with an antibiotic marker suitable for transformation. To create unmarked mutants, the two-step process of Sung et al. (42) using a Janus kanamycin cassette was followed.

### *hsdS* quantification.

Primers [6-FAM]-AMRE74L and AMRE59 (Table S1) were used to PCR amplify a 4.2-kb fragment from single colonies. Amplification was performed in a 25-μl reaction mixture consisting of 0.75 μl 10 mM AMRE74L forward primer, 0.75 μl 10 mM AMRE59 reverse primer, 19.85 μl distilled water (dH_2_O), 2.25 μl 11.1× buffer (for the recipe, see reference 43), 0.2 μl Kapa *Taq* (5 U/μl) (Kapa Biosystems, UK), 0.15 μl Tris (pH 8.8), 0.05 μl *Pfu* (2.5 U/μl), and 1 μl resuspended cells or 1 single colony as the template. The PCR requires a minimum of 100 pg DNA (Table S2). All PCRs were performed as follows: a denaturation step at 95°C for 5 min, followed by 40 cycles of 1 min of denaturation at 95°C, 1 min of annealing at 68°C, and 5 min of extension at 68°C, with a final extension step for 10 min at 68°C. A total of 10 to 15 μl of the PCR product was digested according to the manufacturer’s instructions, using 1 U DraI (New England Biolabs, UK), 2 U PleI (New England Biolabs, UK), and 1× CutSmart buffer (New England Biolabs, UK) in a total volume of 20 μl. Following digestion, each 6-carboxyfluorescein (FAM)-labeled SpnIII variant has a unique size (Table S3) that can be distinguished by capillary electrophoresis on an ABI prism gene analyzer (Applied Biosystems, USA). Data received from the ABI Prism gene analyzer were analyzed using Peak Scanner v1.0 software. All experiments to determine PV at the *spnIII* locus were done by using colonies grown overnight on BHIA with 3% horse blood. For each experiment, a minimum of 10 single colonies were picked directly into the PCR mixture. All colony data are presented as means with standard deviations (SD). The D39 stock maintained by our laboratory predominantly expresses *hsdSE* as its active *hsdS* gene; therefore, colonies not founded by *hsdSE* cells were excluded from the analysis.

### Extraction of S. pneumoniae genomic DNA.

One milliliter of exponentially growing cells (OD_600_ of ∼0.2) was centrifuged at 13,000 rpm, at room temperature, for 2 min to pellet cells. Cells were then washed in 600 μl cell wash buffer (0.15 M NaCl, 0.015 M trisodium citrate, dH_2_O) and centrifuged at 6,000 rpm, at room temperature, for 5 min. After the washing, cells were resuspended in 450 μl of lysis buffer (0.1% sodium deoxycholate [DOC], 0.01% sodium dodecyl sulfate [SDS], Tris-EDTA [TE] [pH 8]) and incubated at 37°C for 10 min, or until turbidity had cleared. A total of 1 mg/ml of proteinase K was added, and samples were incubated at 60°C for 60 min. The Genomic DNA Clean & Concentrator kit (Zymo Research, USA) was then used to extract DNA according to the manufacturer’s instructions. Where required, 1 mg/ml RNase was added to the sample and incubated for 20 min at 37°C. Following extraction, DNA was stored at −20°C.

### RNAseq analysis.

For gene expression analysis, pneumococcal strains were grown to an OD_590_ of 0.15 to 0.18 in triplicate. Five milliliters of cells was added to 1 ml of an ice-cold 95% ethanol–5% phenol solution, before centrifugation at 4,000 rpm for 10 min. The supernatant was removed, and the pellets were stored at −80°C until processing. RNA was extracted using the Maxwell 16 LEV simplyRNA cells kit (Promega, USA) and the Maxwell 16 LEV instrument (Promega, USA). The manufacturer’s protocol was followed from step 4, and the manufacturer’s lysis steps (steps 1 to 3) were replaced with the following protocol to improve cell lysis: pellets were resuspended in 50 μl TE with 3 mg/ml lysozyme and incubated at 37°C for 10 to 20 min. RNAseq analysis was done by using a MiSeq desktop processor (Illumina, USA) and the ScriptSeq complete kit (bacteria) (CamBio, UK), which includes an rRNA depletion step. Raw data were trimmed using Trimmomatic-0.32 and aligned using BWA-Mem and samtools. Expression data were generated using Rockhopper v2.0.3 (44) with D39 as the reference genome (GenBank accession number NC_008533.2).

### UV sensitivity assay.

Cells were grown to an OD_590_ of ∼0.2 in 50 ml TSB. Cultures were centrifuged at 4,000 rpm for 10 min to pellet cells. Cells were resuspended in 20 ml of a 0.9% NaCl solution. A 10-fold serial dilution of cells was plated prior to UV exposure to determine total cell numbers. Cells in 0.9% NaCl were placed in a sterile 90-mm petri dish and exposed to a UV source producing 10 J/min for 45 s. A 10-fold serial dilution of UV-exposed cells was then plated to determine the number of surviving cells. To determine the impact of UV on SpnIII PV, a minimum of 10 single colonies grown for 18 h at 37°C with 5% CO_2_ were selected and used as the DNA template in the *hsdS* quantification protocol described above.

### Protein pulldown and mass spectrometry.

D39 was grown as described above for the transformation protocol in a 50-ml culture. Bacterial cultures were harvested by centrifugation, washed three times with ultrapure water, and frozen at −80°C for a minimum of 1 h. Cells were freeze-thawed a total of 3 times on ice. Cells were resuspended in 1 ml THES buffer (50 mM Tris-HCl [pH 7.5], 10 mM EDTA, 20% sucrose, 140 mM NaCl) and sonicated on ice in 10-s pulses, separated by 1 min.

To test the binding of pneumococcal proteins to the *hsdS* repeats, a synthetic DNA region of 357 bp (Thermo Fisher) containing two copies of the 15-bp IR and 132 bp of the 333-bp IR was amplified and 5′ biotinylated (Btn) with primers [BTN]-AMRE05 and IVPD1 (Table S1). A PCR-amplified fragment of the *pncA* gene, containing no *hsdS* repeats, was used as a control to detect nonspecific binding. The PCR product was concentrated with Amicon/Microcon centrifugal filter (Millipore) columns so that the final concentration of the probe was 200 to 450 ng/μl and then incubated 3 times for 20 min at room temperature with prewashed streptavidin magnetic beads (Pierce) to allow binding. The magnetic beads were prewashed 3 times with 2× binding/washing buffer (10 mM Tris-HCl [pH 7.5], 1 mM EDTA, 2 M NaCl). The probe-bead complex was then washed 3 times with TE and twice with BS-THES (44.3% THES buffer, 20% BS buffer, 35.7% nuclease-free water). To allow binding of the bacterial proteins to the DNA probe, the supernatant of D39 was incubated with the probe-bead complex at room temperature for 1 h. After five washes with BS-THES, the protein-DNA complex was eluted with SDS-PAGE reducing sample buffer, and the proteins were separated by SDS-PAGE using Bis-Tris and Tris-acetate polyacrylamide gels (Invitrogen) and then stained with Coomassie blue, according to standard procedures. The gel was then submitted for mass spectrometry analysis on an LTQ-Orbitrap-Velos-ETD-SN03106B instrument.

## Supplementary Material

Supplemental file 1
