# Supplementary material for: Recombination of the Phase-Variable spnIII Locus Is Independent of All Known Pneumococcal Site-Specific Recombinases
Source: J Bacteriol. 2019 Jul 10;201(15):e00233-19. doi: 10.1128/JB.00233-19 (PMC6620402; doi:10.1128/JB.00233-19)
Supplement: Supplemental file 1 [file JB.00233-19-s0001.pdf]

Supplementary Materials

Recombination of the phase variable *spnIII* locus is independent of all known pneumococcal site-specific recombinases

M De Ste Croix, Y Chen, I Vacca, A S Manso, C Johnston, P Polard, M J Kwun, S D Bentley, N J Croucher, C D Bayliss, R D Haigh, M R Oggioni

Table S1: Primers used in this study

Primer	Sequence	Use
[FAM]AMRE074L	[FAM]GGAAACTGAGATATTTCGTGGTGATGATGGGA	*hsdS* Quantification
AMRE059	CCTGATCGAGCGGAAGAATATTTCTGCCGAGGTTGCC	*hsdS* Quantification
IF100	GCTCTAGAACTAGTGGATC	Spectinomycin *aad9* cassette
IF101	TTCCCTTCAAGAGCGATAC	Spectinomycin *aad9* cassette
MD014	GTTTGATTTTTAATGGATAATGTGATATAATCT	Janus *aphIII-rpsL* cassette
MD015	GGCCCCTTTCCTTATGCTTTTG	Janus *aphIII-rpsL* cassette
VM017	TTATAAAAGCCAGTCATTAGGCC	Chloramphenicol *cat* cassette
VM018	ATGAAAATTTGTTTGATTTTTAATGG	Chloramphenicol *cat* cassette
MD001	GGTGTTAGAATTATACGTGGTGG	*creX*
MD003	TACTGGAACAAGTTATCCTGCAATCAAT	*creX*
MD013	TCCATTAAAAATCAAACAACATTAAATAGTACCAGTATCTCCG	*creX* Janus *aphIII-rpsL* cassette
MD037	CATAAGGAAAGGGGCCCAGACCATTGATTACATTTCTGAGC	*creX* Janus *aphIII-rpsL* cassette
MD038	GCTTTCTTCAAATGTTAATTCAAAATC	*creX* Unmarked
MD039	GATTTTGAATTAACATTTGAAGAAAGC	*creX* Unmarked
MD040	GTCCAGATGTACTATTCTAGTTTC	*xerS::aad9*

MD041	CTAGTTCTAGAGCTCATATTCCTTAAGGTATTCGT ACAGG	*xerS::aad9*
MD042	CTTGAAGGGAAGAATGCTCTGGATAGTTTATGAT TTTG	*xerS::aad9*
MD043	CGAAATCAATCGCATCAAGGATAC	*xerS::aad9*
MD066	GGCTCATCCGGAATAGAGGC	SPD_0921*::aad9*
MD074	GATCCACTAGTTCTAGAGCCCTTCAATAGACTTG CCAGAC	SPD_0921*::aad9*
MD075	GTATCGCTCTTGAAGGGAAGGACAATGATGAACA AGTTGAATG	SPD_0921*::aad9*
MD069	GAATATCCTTCGATCACTCTATC	SPD_0921*::aad9*
MD095	CAGGTAACTCTGGCGGCC	*spoJ::aad9*
MD096	GATCCACTAGTTCTAGAGCCATGATTCTACACTA ACACATC	*spoJ::aad9*
MD097	GTATCGCTCTTGAAGGGAAGCAAACTATCACAAG CCC ATG	*spoJ::aad9*
MD098	GATAGCAAGAATTTTCCACAAGC	*spoJ::aad9*
MD099	GAGGTCAATGGTCGTCTTGTC	*xerD::aad9*
MD105	GATCCACTAGTTCTAGAGCCATCCTTATCAGCTC AAGTCTTAC	*xerD::aad9*
MD106	GTATCGCTCTTGAAGGGAAGGAATTGACAGACAA GCCCTG	*xerD::aad9*
MD102	CAAGGAGTTTAGCCGATGATTG	*xerD::aad9*
MD107	CCCAGGATACGGATGGTATC	*recU::aad9*
MD108	GTATCGCTCTTGAAGGGAACAAGAAACCTACTTA TTGCCGGC	*recU::aad9*
MD109	GATCCACTAGTTCTAGAGCTGGATAGTTGACCAT AATTCTCC	*recU::aad9*
MD110	CTGCTGGTAATCTCTTAACTGC	*recU::aad9*
MD128	GAGGCGAATCAAGCGCTGG	*recA::cat*
MD129	GGTGGCTGCTACTGCTTCC	*recA::cat*
uvrA1	TTACGAGTTGATTGATGCAGTTGAC	*uvrA::aphIII*
Janus-uvrA2	TTATCCATTAAAAATCAAACCTTGCATCTTTGTTC TTTCTAGTCC	*uvrA::aphIII*
Janus-uvrA3	AAAGCATAAGGAAAGGGGCCGAAGCCAGCTATA CAGGACAC	*uvrA::aphIII*
uvrA4	AGATAAGCCAGATGCTCCCAAC	*uvrA::aphIII*

[BTN]AMRE05	TGAATCAGTCGAAGTTTTACTTGAA	Protein pull-down
IVPD1	TGGTCAAACTGACTACCTGCT	Protein pull-down

Table S2 – active *hsdS* quantification on serial dilutions of chromosomal DNA

	Quantity of DNA (pg)	SpnIIIA	SpnIIIB	SpnIIIC	SpnIIID	SpnIIIE	SpnIIIF	Total peak area
**PCR**	100	3,5	2	0	0,7	93,8	0	15094
	10	2,3	0	0	0	97,7	0	7704
	1	0	0	0	0	100	0	2129
	0.1	0	0	0	0	100	0	312
	0.01	0	0	0	0	100	0	90
	0.001	-	-	-	-	-	-	-

Table S3 – FAM-labelled fragment sizes of each active *hsdS* gene following digestion with DraI and PleI

hsdS gene	Enzyme Site	Fragment Size (bp)
SpnIII A	PleI	1186
SpnIII B	DraI	1109
SpnIII C	DraI	1090
SpnIII D	PleI	1167
SpnIII E	DraI	1052
SpnIII F	DraI	1032

Table S4: WT strains used in this study

Strain	Serotype	Sequence type	Reference
D39	2	ST595	(1)
SP3-BS71	3	ST180	(2)
BHN35	3	ST180	(3)
TIGR4	4	ST205	(4)
BHN191	6B	ST138	(5)
BHN418	6B	ST138	(5)
MLV-016	11A	ST62	(6)
AP200	11A	ST62	(7)
SP11-BS70	11A	ST62	(2)
INV200	14	ST9	(8)
SP14-BS69	14	ST124	(2)
BHN100	19F	ST162	(9)
CBR206	19F	ST179	(10)
LgST215	19F	ST179	(10)
G54	19F	ST63	(11)

Table S5: Mean percentage of recombination by repeat in single recombinase mutants

	333bp Repeat		85bp Repeat		15bp Repeat	
	Mean(SD) %	P value	Mean(SD) %	P value	Mean(SD) %	P value
D39 WT	16.8±5.4	-	1.5±0.8	-	4.1±1.9	-
D39 ΔSPD_0921	17.9±1.9	NS	2.5±0.4	NS	2.1±3.0	NS
D39 *ΔxerS*	16.1±2.5	NS	2.0±0.7	NS	2.3±1.7	NS
D39 *ΔxerD*	19.7±5.2	NS	2.2±1.4	NS	2.4±1.3	NS

Table S6: Proteins identified by Mass Spec using a 1.2kB synthetic hsdS DNA fragment.

Protein	SPD number	Function
AgrR	SPD_1904	transcriptional regulator of arginine metabolism
AliA	SPD_0334	oligopeptide ABC transporter, oligopeptide-binding protein
DnaK	SPD_0460	molecular chaperone
FabZ	SPD_0387	3-hydroxyacyl-[acyl-carrier-protein] dehydratase
GlmU	SPD_0874	UDP-N-acetylglucosamine pyrophosphorylase
GyrA	SPD_1077	DNA gyrase subunit A
GyrB	SPD_0709	DNA gyrase subunit B
LigA	SPD_1001	DNA ligase
ParC	SPD_0748	Topoisomerase IV subunit A
ParE	SPD_0746	Topoisomerase IV subunit B
RecG	SPD_1507	ATP-dependent DNA helicase
RnhB	SPD_1020	ribonuclease HII
RpoE	SPD_0441	DNA-directed RNA polymerase D subunit, putative
SPD_1378	SPD_1378	Conserved hypothetical protein
ThiI	SPD_0777	tRNA uracil 4-sulfurtransferase
UvrA	SPD_0176	ATPase and DNA-binding protein part of uvrABC repair complex

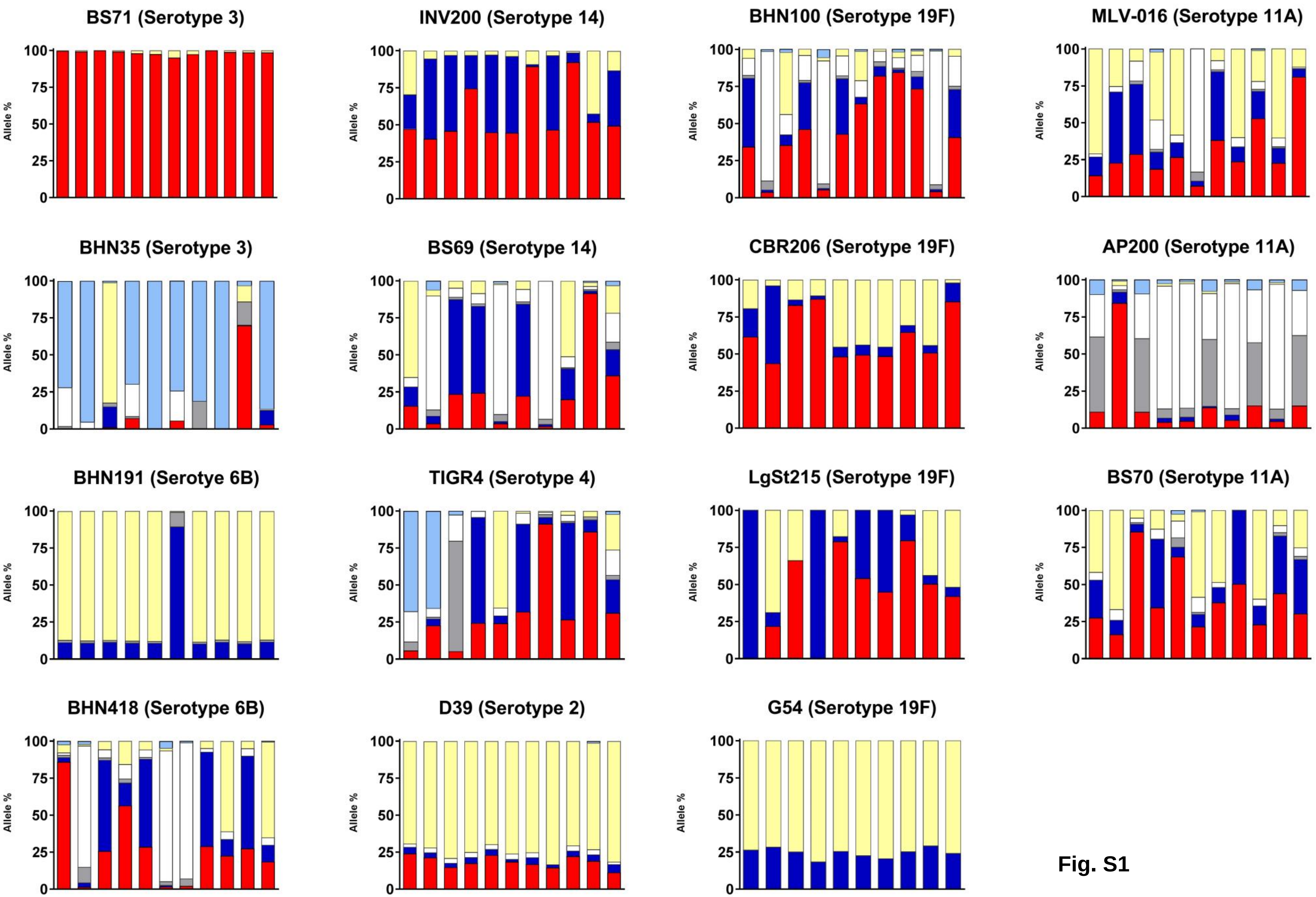
BS71 (Serotype 3)
INV200 (Serotype 14)
BHN100 (Serotype 19F)
MLV-016 (Serotype 11A)
BHN35 (Serotype 3)
BS69 (Serotype 14)
CBR206 (Serotype 19F)
AP200 (Serotype 11A)
BHN191 (Serotye 6B)
TIGR4 (Serotype 4)
LgSt215 (Serotype 19F)
BS70 (Serotype 11A)
BHN418 (Serotype 6B)
D39 (Serotype 2)
G54 (Serotype 19F)
Allele %
0
25
50
75
100

Fig. S1

Figure S1 – *spnIII* TRD recombination in different WT background. Each graph shows the distribution of active *hsdS* genes in 15 different wildtype *S. pneumoniae* strains. Each bar represents a single colony and each colour represents an active *hsdS* gene as follows; *hsdSA* (red), *hsdSB* (dark blue), *hsdSC* (grey), *hsdSD* (white), *hsdSE* (yellow) and *hsdSF* (light blue). Strain G54 lacks TRD's 1.2 and 2.1 so cannot generate an active *hsdSA, D, C* or *F.* Strain BHN191 lacks TRD 2.1 so cannot generate an active *hsdSA* or *D,* all other strains show recombination on all three inverted repeats. There is no correlation between serotype and/or sequence type and *spnIII* recombination.

Fig. S2

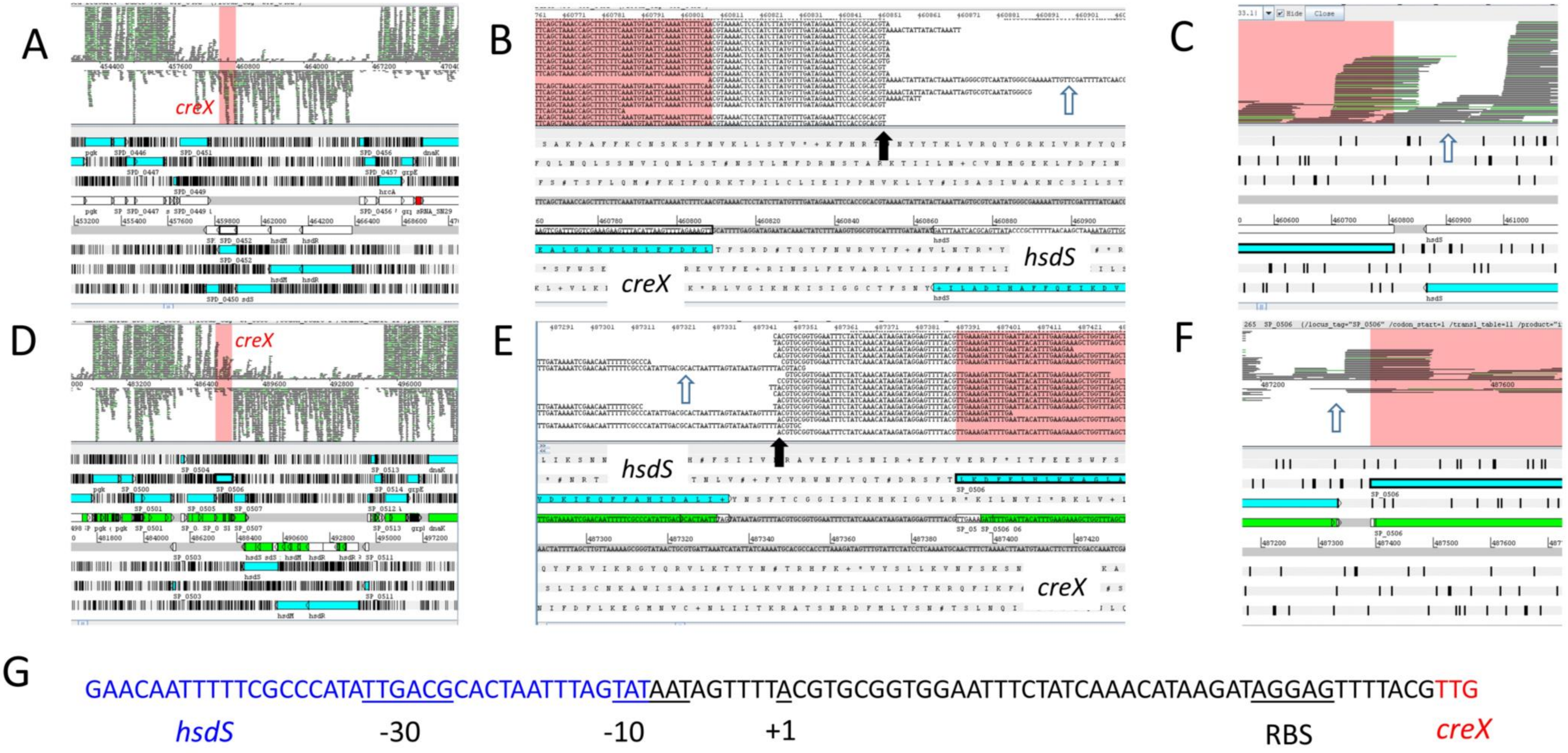

Figure S2 – differential gene expression at the *spnIII* locus. RNAseq data of strains predominantly expressing a single active *hsdS* gene have been plotted by strand. Due to different *spnIII* orientations in the genome annotations RNA from

hsdSA cells has been plotted on D39 (A) and *hsdSB* cells plotted on TIGR4 (D). The *creX* gene is in red in all panels. The *creX* transcript is independent of the *hsdS* transcript and starts in the intergenic region at the same nucleotide in *spnIIIA* and *spnIIIB* (filled arrow) (B and E), indicating that the *creX* promoter does not alter with orientation. The most likely promoter consensus (underlined) is within the *hsdS* coding region (shown in blue) (G). RNAseq reads spanning from *hsdS* to *creX* are few but more frequent in *spnIIIA* (n=18) than in *spnIIIB* (n=8) (open arrows in B, C, E and F

Supplementary references

1. Lanie J a., Ng WL, Kazmierczak KM, Andrzejewski TM, Davidsen TM, Wayne KJ, Tettelin H, Glass JI, Winkler ME. 2007. Genome sequence of Avery's virulent serotype 2 strain D39 of Streptococcus pneumoniae and comparison with that of unencapsulated laboratory strain R6. J Bacteriol 189:38–51.
2. Hiller NL, Janto B, Hogg JS, Boissy R, Yu S, Powell E, Keefe R, Ehrlich NE, Shen K, Hayes J, Barbadora K, Klimke W, Dernovoy D, Tatusova T, Parkhill J, Bentley SD, Post JC, Ehrlich GD, Hu FZ. 2007. Comparative genomic analyses of seventeen Streptococcus pneumoniae strains: Insights into the pneumococcal supragenome. J Bacteriol 189:8186–8195.
3. Croucher NJ, Mitchell AM, Gould KA, Inverarity D, Barquist L, Feltwell T, Fookes MC, Harris SR, Dordel J, Salter SJ, Browall S, Zemlickova H, Parkhill J, Normark S, Henriques-Normark B, Hinds J, Mitchell TJ, Bentley SD. 2013. Dominant Role of Nucleotide Substitution in the Diversification of Serotype 3 Pneumococci over Decades and during a Single Infection. PLoS Genet 9 (10).
4. Tettelin H, Nelson KE, Paulsen IT, Eisen JA, Read TD, Scott P. 2001. Complete Genome Sequence of a Virulent Isolate of Streptococcus pneumoniae. Science (5529) 293:498–506.
5. Browall S, Norman M, Tångrot J, Galanis I, Sjöström K, Dagerhamn J, Hellberg C, Pathak A, Spadafina T, Sandgren A, Bättig P, Franzén O, Andersson B, Örtqvist Å, Normark S, Henriques-Normark B. 2014. Intraclonal variations among streptococcus pneumoniae isolates influence the likelihood of invasive disease in children. J Infect Dis 209:377–388.

6. Bidossi A, Mulas L, Decorosi F, Colomba L, Ricci S, Pozzi G, Deutscher J, Viti C, Oggioni MR. 2012. A functional genomics approach to establish the complement of carbohydrate transporters in Streptococcus pneumoniae. PLoS One 7.
7. Camilli R, Bonnal RJP, Del Grosso M, Iacono M, Corti G, Rizzi E, Marchetti M, Mulas L, Iannelli F, Superti F, Oggioni MR, De Bellis G, Pantosti A. 2011. Complete genome sequence of a serotype 11A, ST62 Streptococcus pneumoniae invasive isolate. BMC Microbiol 11:25.
8. Donati C, Hiller NL, Tettelin H, Muzzi A, Croucher NJ, Angiuoli S V, Oggioni M, Dunning Hotopp JC, Hu FZ, Riley DR, Covacci A, Mitchell TJ, Bentley SD, Kilian M, Ehrlich GD, Rappuoli R, Moxon ER, Masignani V. 2010. Structure and dynamics of the pan-genome of Streptococcus pneumoniae and closely related species. Genome Biol 11:R107.
9. Fälker S, Nelson AL, Morfeldt E, Jonas K, Hultenby K, Ries J, Melefors Ö, Normark S, Henriques-Normark B. 2008. Sortase-mediated assembly and surface topology of adhesive pneumococcal pili. Mol Microbiol 70:595–607.
10. Tavares DA, Simões AS, Bootsma HJ, Hermans PW, de Lencastre H, Sá-Leão R. 2014. Non-typeable pneumococci circulating in Portugal are of cps type NCC2 and have genomic features typical of encapsulated isolates. BMC Genomics 15:863.
11. Dopazo J, Mendoza A, Herrero J, Cadara F, Humbert Y, Friedli L, Guerrier M, Grand-Schenk E, Gandin C, de Francesco M, Polissi A, Buell G, Feger G, Garcia E, Peitsch M G-BJ. 2001. Annotated draft genomic sequence from a Streptococcus pneumoniae type 19F clinical isolate. Microb drug Resist 7:99–

125.
